# Congenital Malformation of the Cornea and Sclerotica

**Published:** 1831

**Authors:** 


### Congenital Malformation of the Cornea and Sclerotica.

A servant, æt. 22, has a small excrescence on the left eye, arising from the cornea and sclerotica, at its temporal side; its surface white, smooth, convex; in its centre a small depression from which strong black hairs arise, which incline downwards so as to hang over the lower lid; it is firm to the touch, and appears to be covered by the sclerotic conjunctiva; the surrounding cornea and sclerotica preserve their natural structure. He says that the blemish was congenital, and that the hairs began to plague him only a few years ago. The man usually calls on Mr. Middlemore once in every six weeks, in order to have the hairs removed. Mr. M. intends to apply the nitrate of silver to the cavities containing the bulbs of the largest hairs, when he next extracts them, and if this fails to dissect them out, if it can be done without danger of penetrating the globe. The hairs are of the same colour and have the same inclination as those of the lower eyelid. This is the only instance of such a congenital malformation which Mr. Middlemore has witnessed.

In a patient with hernia we remarked a deficiency of the circle of the iris at its inferior part. The pupil was of course oblong from above downwards; vision was very imperfect with that eye. The same individual had all the appearances of the blue disease, though not in an aggravated degree, so that there seemed to be a tendency to imperfection in her formation, a circumstance not uncommon.—*Ibid.*

